# Earnings of US Physicians With and Without Disabilities

**DOI:** 10.1001/jamahealthforum.2023.3954

**Published:** 2023-12-01

**Authors:** Mihir Kakara, Atheendar S. Venkataramani

**Affiliations:** 1 Department of Neurology, Perelman School of Medicine, University of Pennsylvania, Philadelphia; 2 Leonard Davis Institute of Health Economics, University of Pennsylvania, Philadelphia; 3 Department of Medical Ethics and Health Policy, Perelman School of Medicine, University of Pennsylvania, Philadelphia

## Abstract

This cross-sectional study uses American Community Survey data to assess disability earnings gaps for physicians between 2005 and 2019.

## Introduction

Workers with disabilities earn less than workers without disabilities across industries.^1^ These earnings disparities have not been well studied among physicians. Potential disability earnings gaps in medicine are important to characterize as more individuals with disabilities enter medicine, facing significant challenges in medical education^2^ and higher risks of mistreatment at work.^3^ Furthermore, as the share of physicians in older age groups grows,^4^ disability incidence increases.

## Methods

This cross-sectional study analyzed data from the 2005 to 2019 American Community Survey (ACS).^5^ Institutional review board review was not required given the use of public deidentified data. We followed the STROBE reporting guideline for cross-sectional studies. Physicians aged 35 to 65 years were identified by self-reported occupation as physician or surgeon. Disability status was defined as answering yes to any of 6 disability types queried in the ACS (eMethods in Supplement 1). We used data on employment status, hours and weeks worked, and annual personal earned income. We used linear regression models to examine differences in logged annual and hourly personal earned income and total hours worked by disability status, adjusting for age, sex, self-reported race and ethnicity, state-metropolitan area fixed effects, and survey year, along with subgroup analyses of annual income by age group and disability type. ACS collected surgical specialty from 2018 onwards. Survey weights were used for all analyses, which were conducted in April 2023 using Stata version 17.0 (StataCorp). Significance was set at 2-sided *P* < .05.

## Results

The study cohort included 92 469 physicians, of whom 1953 (weighted, 2.0%) reported having a disability (Table 1). In adjusted analyses of employed physicians, annual earned income was 20.8% (95% CI, −25.2% to −16.3%; *P* < .001) lower and hourly earned income was 13.3% (95% CI, −17.3% to −9.4%; *P* < .001) lower among physicians with disabilities. Physicians reporting disabilities worked 110 hours per year less on average compared with those without disabilities. Estimated differences in annual income by disability status were similar after adjusting for hours worked and surgical specialty status and across age groups; estimates were much larger for disabilities affecting cognitive function, ambulation, independent living, and self-care than vision or hearing (Table 2).

**Table 1.** Characteristics of US Physicians by Disability Status, 2005 to 2019^a^

Characteristic	No. (%)		*P* value
	No disability (n = 90 516)	Any disability (n = 1953)^b^	
Age, median (SD), y	48 (8.7)	55 (8.6)	<.001
Age group, y			
35-44	32 804 (99.0)	323 (1.0)	<.001
45-54	30 437 (98.3)	577 (1.7)	
55-65	27 275 (96.5)	1053 (3.5)	
Sex			
Female	30 447 (98.0)	655 (2.0)	<.001
Male	60 069 (98.1)	1298 (1.9)	
Race and ethnicity			
American Indian or Alaska Native	174 (91.2)	13 (8.8)	<.001
Black/African American	3696 (97.9)	81 (2.1)	
Chinese	3562 (98.9)	43 (1.1)	
Hispanic	5063 (97.7)	120 (2.3)	
Cuban	793 (98.1)	19 (1.9)	
Mexican	1331 (96.6)	50 (3.4)	
Puerto Rican	728 (97.4)	16 (2.6)	
Other Hispanic^e^	2211 (98.3)	35 (1.7)	
Not Hispanic	85 543 (98.1)	1833 (1.9)	
Japanese	483 (98.9)	8 (1.1)	
Pacific Islander	36 (90.8)	2 (9.2)	
South Asian	7931 (98.9)	93 (1.0)	
White	68 334 (97.9)	1590 (2.2)	
2 Races	1436 (98.1)	34 (1.9)	
≥3 Races	127 (94.6)	9 (5.4)	
Other Asian^c^	4032 (98.7)	63 (1.3)	
Other race, NEC^d^	705 (98.4)	17 (1.6)	
Disability type			
Sensory^f^	NA	1074 (55.4)	NA
Hearing^f^	NA	642 (39.9)	NA
Vision^f^	NA	296 (19.8)	NA
Ambulatory	NA	476 (24.2)	NA
Self-care	NA	146 (7.5)	NA
Cognitive	NA	133 (6.5)	NA
Independent living	NA	124 (6.3)	NA
Labor force participation			
Not in labor force	1016 (92.3)	87 (7.8)	<.001
Among employed physicians			
Usual hours worked per week, mean (SD)^g^	51.05 (14.82)	48.71 (16.83)	<.001
Weeks worked last year^g^			
1-13	447 (93.5)	30 (6.5)	<.001
14-26	702 (96.7)	32 (3.4)	
27-39	1261 (96.6)	46 (3.4)	
40-47	4133 (97.6)	107 (2.4)	
48-49	4406 (97.9)	89 (2.2)	
50-52	78 277 (98.2)	1542 (1.8)	
Annual earned income,^h^ mean (SD), $^g^^,^^i^	131 287.28 (79 175.34)	113 186.27 (78 654.66)	<.001
Hourly earned income,^j^ mean (SD), $^g^	53.51 (32.79)	48.48 (32.16)	<.001

Abbreviations: NA, not applicable; NEC, not elsewhere classified.

^a^ Unadjusted and weighted. Weighting was done for means and SDs for continuous variables and for percentages only for categorical variables.

^b^ Defined as answering yes to any of the 6 disability questions in the American Community Survey (ACS) related to vision, hearing, cognitive, ambulatory, self-care, or independent-living.

^c^ Includes Bhutanese, Burmese, Cambodian, Filipino, Hmong, Indonesian, Korean, Laotian, Malaysian, Mongolian, Nepalese, Taiwanese, Thai, Vietnamese, and other Asian ethnicities not included in above categories.

^d^ All other races not elsewhere classified in the ACS.

^e^ Includes Argentinian, Bolivian, Chilean, Colombian, Costa Rican, Dominican, Ecuadorian, Guatemalan, Honduran, Nicaraguan, Panamanian, Paraguayan, Peruvian, Salvadoran, Spaniard, Uruguayan, Venezuelan, and other Hispanic ethnicities not included in above categories.

^f^ Includes vision or hearing disability or both. This was combined for all years as ACS started asking separate questions for vision and hearing disability only from 2008. Vision and hearing disability prevalence are reported separately only for years 2008-2019, and hence do not add up to 100%.

^g^ Includes only physicians who reported being employed.

^h^ Annual earned income denotes income earned from wages or a person’s own business or farm for the previous year. It is the total of annual wages and net preincome tax self-employment income from a business, professional practice, or farm.

^i^ All values adjusted to 2011 US dollars.

^j^ Hourly earned income calculated by dividing annual earned income by total hours worked in the year. Total hours worked is calculated by multiplying hours worked per week and weeks worked in the year.

**Table 2.** Differences in Earnings and Total Hours Worked for Employed US Physicians With vs Without a Disability^a^

Variable	Adjusted models		Models additionally adjusting for total hours worked	
	Estimate [SE] (95% CI)	*P* value	Estimate [SE] (95% CI)	*P* value
**Overall analysis**				
Difference in annual earned income,^b^ percentage points^c^	−20.8 [0.02] (−25.2 to −16.3)	<.001	−18.3 [0.02] (−22.5 to −14.2)	<.001
Difference in total hours worked (annual)^d^	−109.7 [25.01] (−158.7 to −60.7)	<.001	NA	NA
Difference in hourly earned income,^e^ percentage points^c^	−13.3 [0.02] (−17.3 to −9.4)	<.001	−15.4 [0.02] (−19.4 to −11.4)	<.001
**Subgroup analyses for years 2018 and 2019**				
Difference in annual earned income,^b^ percentage points^c^				
Model without surgical specialty^f^	−11.5 [0.06] (−22.9 to −.0000173)	.05	−11.4 [0.05] (−21.8 to −1.1)	.03
Model with surgical specialty^f^	−11.6 [0.06] (−22.7 to −0.5)	.04	−11.6 [0.05] (−22.2 to −0.5)	.04
**Subgroup analysis by age group**				
Difference in annual earned income,^b^ percentage points^c^				
Age 35-44 y	−13.7 [0.05] (−24.3 to −3.2)	.01	−13.4 [0.05] (−23.6 to −3.3)	.01
Age 45-54 y	−23.2 [0.04] (−31.7 to −14.7)	<.001	−21.8 [0.04] (−29.7 to −13.9)	<.001
Age 55-65 y	−22.1 [0.03] (−27.9 to −16.3)	<.001	−16.9 [0.03] (−22.2 to −11.8)	<.001
**Subgroup analyses by disability type compared with those without disability**				
Difference in annual earned income,^b^ percentage points^c^				
Cognitive	−40.4 [0.09] (−57.8 to −22.9)	<.001	−36.2 [0.08] (−52.0 to −20.4)	<.001
Ambulatory	−40.5 [0.05] (−49.4 to −31.5)	<.001	−36.0 [0.04] (−44.4 to −27.6)	<.001
Independent living	−45.0 [0.12] (−67.7 to −22.4)	<.001	−37.4 [0.11] (−58.4 to −16.4)	<.001
Self-care	−43.6 [0.09] (−61.1 to −26.2)	<.001	−33.7 [0.08] (−49.4 to −18.1)	<.001
Vision	−10.7 [0.06] (−21.8 to 0.3)	.06	−7.6 [0.15] (−17.9 to 2.8)	.15
Hearing	−2.44 [0.03] (−8.8 to 3.9)	.45	−3.7 [0.23] (−9.9 to 2.4)	.23

Abbreviation: NA, not applicable.

^a^ All outcomes were analyzed for employed physicians aged 35-65 years, using linear regression models with robust SEs. Models were adjusted for fixed effects for age (individual years), sex (male, female), race (Black/African American, White, and other), ethnicity (Hispanic, not Hispanic), state and metropolitan area, and survey year.

^b^ Annual earned income denotes income earned from wages or a person’s own business or farm for the previous year. It is the total of annual wages and net preincome tax self-employment income from a business, professional practice, or farm.

^c^ Because logged income was used as the dependent variable, estimated earnings disparities can be expressed as percent differences (coefficient multiplied by 100).

^d^ Total hours in a year worked is calculated by multiplying hours worked per week and weeks worked in the year.

^e^ Hourly earned income calculated by dividing annual earned income by total hours worked in the year. Total hours worked is calculated by multiplying hours worked per week and weeks worked in the year.

^f^ The American Community Survey started dividing physicians into “surgeons” and “other physicians” from 2018. For years 2018 and 2019, total number of surgeons in these 2 years was 838, with 820 reporting no disability, and 18 reporting a disability. The total number of nonsurgeons was 12 568, with 12 331 reporting no disability and 237 reporting a disability.

## Discussion

Physicians reporting disabilities had significantly lower earnings than physicians without disabilities. These results should motivate new efforts to better characterize these disparities and address critical data gaps. For example, data on age at disability onset, degree of disability, and longitudinal earnings trajectories before and after disability onset will be crucial for understanding how income may vary across physicians and over time as well as underlying mechanisms.

Similarly, larger physician data sets that already collect subspecialty and income data should collect disability data to characterize the extent to which specialty sorting may explain our findings. The increasing disability prevalence with age suggests many disabilities were acquired after choosing a specialty. If subspecialty-based income gaps are found to exist, physicians with disabilities may continue to face structural barriers to entering and staying in high-paying or procedural specialties, even though qualitative work has documented successful integration of physicians with disabilities into procedural specialties.^2^

In addition, collecting data on potential discrimination faced by physicians with a disability is important. Employer discrimination may be a key basis of disability-based income disparities.^1^

As a population-based census survey, the ACS has advantages of a large sample size, detailed income measures, and potentially less susceptibility to stigma-related underreporting of disability. Our study also has potential limitations. First, physician incomes may be underreported in the ACS.^5^ How this underreporting biases our results is not clear. Second, the disability prevalence (2.0%) in our sample is lower than a previously reported estimate (3.1%).^6^ This discrepancy may be explained by differences in sample sizes, sample age cutoffs, and disability definition.

## Notes

Supplement 1. **eMethods.** Data Source, Identification of Sample, Covariates, and Outcome Measures
eReferences

Supplement 2.
Data Sharing Statement
