# Supplementary material for: Earnings of US Physicians With and Without Disabilities
Source: JAMA Health Forum. 2023 Dec 1;4(12):e233954. doi: 10.1001/jamahealthforum.2023.3954 (PMC10692836; doi:10.1001/jamahealthforum.2023.3954)
Supplement: Supplement 1. — eMethods. Data Source, Identification of Sample, Covariates, and Outcome Measures eReferences [file jamahealthforum-e233954-s001.pdf]

Supplemental Online Content

Kakara M, Venkataramani, A. Earnings of US physicians with and without disabilities. *JAMA Health Forum.* 2023;4(12):e233954. doi:10.1001/jamahealthforum.2023.3954

eMethods. Data Source, Identification of Sample, Covariates, and Outcome Measures
eReferences

This supplemental material has been provided by the authors to give readers additional information about their work.

© 2023 Kakara et al. *JAMA Health Forum*.

eMethods

Data Source: The American Community Survey (ACS) is an annual, cross-sectional, national representative survey administered by the U.S. Census Bureau. Data from the ACS is publicly available and was accessed through Integrated Public Use Microdata Series (IPUMS) database.[1]

Identification of sample:
Physicians were identified as those reporting occupation as 'physician or surgeon', which was a single unique code till 2017. From 2018, this was subdivided into 'surgeons' and 'other physicians'. 'Surgeons' included orthopedic surgeon, ophthalmologists, pediatric surgeons, and 'all other surgeons'.

Physicians were identified to have a disability if they answered Yes to any of the standard 6 disability questions in the ACS:

1) Because of a physical, mental, or emotional condition, does this person have serious difficulty concentrating, remembering, or making decisions?
2) Does this person have serious difficulty walking or climbing stairs?
3) Because of a physical, mental, or emotional condition, does this person have difficulty doing errands alone such as visiting a doctor's office or shopping?
4) Does this person have difficulty dressing or bathing?
5) Is this person blind or does he/she have serious difficulty seeing even when wearing glasses?
6) Is this person deaf or does he/she have serious difficulty hearing?

Hearing and vision disability were combined into 'sensory' disability by the ACS till 2007, and separate questions for these were asked only from 2008 onwards.

The lower age cut-off of 35 years was chosen to exclude physicians in training. This has been used in papers that have investigated racial and gender wage gaps among physicians.[2,3] The upper age cut-off of 65 years was chosen since that is around the traditional retirement age, and to make sure to minimize skewing from age-related disabilities.

Covariates:
Race: All the different self-reported race categories as used in the ACS were reported – White, Black/African American, American Indian or Alaska Native, Chinese, Japanese, Other Asian or Pacific Islander, Other race nec (not elsewhere classified), two major race, three or more major races.
Pacific Islanders were separated from the broader 'Other Asian or Pacific Islanders' and included the following: Hawaiian, Hawaiian mixed, Pacific Islander only (CPS), Samoan, Tahitian, Tongan, Other Polynesian (1990), 1+ Other Polynesian race (2000, ACS), Chamorro, Northern Mariana Islander, Palauan, Other Micronesian (1990), 1+ other Micronesian races (2000, ACS), Fijian, 2+ PI races from 2+ PI regions. Among Asians, South Asians were separately reported and included the following: Asian Indian, Bhutanese, Nepalese, Bangladeshi, Pakistani, Sri Lankan. 'Other Asian' were all other Asians except South Asians and Pacific Islanders in the 'Other Asian and Pacific Islander' category in the ACS. These would include Taiwanese, Filipino, Korean, Vietnamese, Bhutanese, Mongolian, Nepalese, Cambodian, Hmong, Laotian,

© 2023 Kakara et al. *JAMA Health Forum*.

Thai, Burmese, Indonesian, Malaysian and other Asians not included in above categories. Categories for Pacific Islanders and South Asians was based on the definitions provided by the Asian Pacific Institute on Gender-based Violence definition.

Ethnicity was reported as Hispanic or Not Hispanic. Among Hispanic, Cuban, Mexican and Puerto Rican ethnicities were reported separately. Other Hispanic included Costa Rican, Guatemalan, Honduran, Nicaraguan, Panamanian, Salvadoran, Argentinian, Bolivian, Chilean, Colombian, Ecuadorian, Paraguayan, Peruvian, Uruguayan, Venezuelan, Spaniard, Dominican and other Hispanic ethnicities not included in above categories.

Both race and ethnicity were self-reported by participants in the ACS.For state-fixed effects, individuals states were identified using the Inter-University Consortium for Political and Social Research (ICPSR) codes in ACS. For metropolitan area fixed-effects, metropolitan areas were identified as either being in a metropolitan area, a mixed metropolitan area or not in a metropolitan area.

Outcome Measures: The primary measures of income used was the annual personal earned income, which is the total of income from wages/salaries and the person's own business. Income was inflation adjusted to 2011 US dollars, and the top and bottom 2.5% values were winsorized to remove outliers. Hourly earned income calculated by dividing annual earned income by total hours worked in the year. Total hours worked as calculated by multiplying hours worked per week and weeks worked in the year.

eReferences:

1. Ruggles S, Flood S, Sobek M, Brockman D, Cooper G, Richards S, Schouweiler M. IPUMS USA: Version 13.0 [dataset]. Minneapolis, MN: IPUMS, 2023. https://doi.org/10.18128/D010.V13.0

2. Ly DP, Seabury SA, Jena AB. Differences in incomes of physicians in the United States by race and sex: observational study. BMJ. 2016 Jun 7;353:i2923. Doi: 10.1136/bmj.i2923. PMID: 27268490; PMCID: PMC4897176.

3. Ly DP. Historical Trends in the Representativeness and Incomes of Black Physicians, 1900-2018. J Gen Intern Med. 2022 Apr;37(5):1310-1312. doi: 10.1007/s11606-021-06745-1. Epub 2021 Apr 19. PMID: 33876377; PMCID: PMC8971221.

4. Asian and Pacific Islander Ethnicities and Regional Grouping. Asian Pacific Institute on Gender-based Violence. https://www.api-gbv.org/resources/census-data-api-identities/ . Accessed September 13, 2023.

© 2023 Kakara et al. *JAMA Health Forum*.
