# Supplementary material for: Earnings of US Physicians With and Without Disabilities
Source: JAMA Health Forum. 2023 Dec 1;4(12):e233954. doi: 10.1001/jamahealthforum.2023.3954 (PMC10692836; doi:10.1001/jamahealthforum.2023.3954)
Supplement: Supplement 2. — Data Sharing Statement [file jamahealthforum-e233954-s002.pdf]

Data Sharing Statement

Kakara. Earnings of US Physicians With and Without Disabilities. *JAMA Health Forum*. Published December 01, 2023. doi:10.1001/jamahealthforum.2023.3954

Data
Data available: No

Additional Information
Explanation for why data not available: The data used in these analyses are publicly available.
